# How Swedish healthcare workers managed the early weeks of the COVID-19 pandemic: sensemaking across organisational, interpersonal and individual levels

**DOI:** 10.1186/s12913-026-15826-y

**Published:** 2026-10-10

**Authors:** Helena Månsson Sandberg, Mats Jong, Miek C. Jong, Erika Wall

**Affiliations:** 1 Department of Health Sciences, Mid Sweden University, Östersund/Sundsvall, Sweden; 2 Department of Community Medicine, National Research Center in Complementary and Alternative Medicine (NAFKAM), UiT, The Arctic University of Norway, Tromsø, Norway

**Keywords:** COVID-19, Crisis, Doctors, Healthcare workers, Nurses, Pandemic, Physicians, Sensemaking, Work situation

## Abstract

**Background:**

In times of crisis the need to make sense of the situation becomes particularly evident. The perspective of sensemaking captures how individuals seek to clarify and understand what is going on when they encounter ambiguous, unexpected or confusing events, such as the COVID-19 pandemic. Sensemaking may occur at different but mutually interrelated organisational, interpersonal and individual levels. During the COVID-19 pandemic, healthcare workers (HCWs) faced multifaceted challenges related to their work. This study aimed to explore how HCWs managed their daily work during the early weeks of the COVID-19 pandemic through the perspective of sensemaking across organisational, interpersonal and individual levels.

**Method:**

The data was collected through individual interviews with 18 nurses and physicians between March and May 2020, and analysed using a reflexive thematic analysis.

**Findings:**

The analysis revealed an overarching pattern of sensemaking as a way of holding clinical practice together throughout uncertainty. At an organisational level, sensemaking included how organisational structures contributed to stability in the organisation of work. The interpersonal level captured how understanding of uncertain circumstances was grounded in a collective understanding wherein HCWs reflected together and in relation to others. The individual level of sensemaking captured HCWs´ capability and commitment to continuing to provide care, despite uncertain circumstances.

**Conclusions/Implications:**

The findings imply that sensemaking at organisational, interpersonal and individual levels is an important consideration for healthcare organisations when facing future crisis or other uncertain events. Clear organizational directives are important for fostering a shared understanding of how uncertain situations should be interpreted and managed in practice. Furthermore, HCWs need opportunities and time for interaction with colleagues, enabling them to collectively construct meaning and maintain a sense of stability when managing uncertainty. It is also important to acknowledge HCWs´ individual knowledge, experiences and capability to sustain clinical practice despite conditions of uncertainty.

**Supplementary Information:**

The online version contains supplementary material available at https://doi.org/10.1186/s12913-026-15826-y.

## Background

At different points in history, societies have faced significant crises that have disrupted and challenged everyday life. A crisis is often characterised as a threatening, low-probability and high-impact event [1], and can come in different forms such as natural disasters, accidents and public health crises [2]. In times of crisis there is an evident need to make sense of the situation [1, 3]. As proposed by Weick, the theoretical perspective of sensemaking captures how individuals seek to clarify and understand what is going on when they encounter ambiguous, unexpected or confusing events [4]. Although sensemaking is particularly evident during crises [3], it is not limited to such events and even minor events can trigger sensemaking [5]. In organisational research, sensemaking captures how individuals within the organisation understand events that in some way violate their expectations [6], such as decisions that lead to changes in the organisation of work [7]. Accordingly, sensemaking can be triggered when events are experienced as sufficiently deviating from expectations, thus prompting individuals to question what is happening and how they should act [6, 8]. The term ´enactment´ is central in this regard and it refers to how individuals shape and give meaning through their actions, which in turn shape and structure their environment [1, 4]. From this perspective, sensemaking is understood as ongoing, with individuals within an organisation interpreting and assigning plausible meaning to their actions and experiences [4, 8]. In so doing, individuals draw on cues from their social context and reflect on unfolding events to construct explanations that make specific situations understandable [8]. The social aspect is significant from the perspective of sensemaking as meanings are often constructed and negotiated through interactions and dialogues with others [4, 9]. Additionally, at an organisational level of sensemaking members construct accounts that enable them to understand their environment and act together [10], which can manifest in organisational circumstances being translated into words and written texts [8]. An example of organisational sensemaking is how rules regulate and structure behaviour in organisations, whereby the combination of rules configures expectations and prescribes the way things are done or should be done [11]. As such, the organisation provides frameworks that guide how situations are interpretated and acted upon [11]. To sum up, in organisations such as workplaces, sensemaking can take place at different but mutually interrelated levels; the organisational level, interpersonal level and individual level [11]. Previous literature on sensemaking in times of crisis provides the understanding that individual sensemaking does not occur in isolation, but in interaction with others and organisational structures [1, 3, 6]. For example, it has been demonstrated that individuals´ sensemaking and actions are shaped by the expectations and values established by management within the organization [1]. Furthermore, in times of crisis the construction of shared meaning in terms of collective commitment, shared identity and shared expectations between individuals within the organisation play an important role. This suggests that individual sensemaking is influenced by the meaning that individuals within an organisation create together [3]. The COVID-19 pandemic is a recent example of a crisis that disrupted our society and created a level of uncertainty rarely experienced in modern times [12]. The COVID-19 pandemic caused mortality [13], lock-downs of societal functions [14], and general uncertainty about how people might be affected by the pandemic and how to respond to it [15]. Consequently, the COVID-19 pandemic led to crisis in many different areas [2], including workplaces and the healthcare system [16]. We now know that healthcare workers (HCWs) faced multifaceted challenges related to their work during the years of the pandemic. Nurses and physicians were at the frontline and needed to rapidly reorganise the provision of care [17–19] in order to maintain high-quality care standards under sub-optimal conditions [20]. Working in uncertain circumstances like these challenged established routines and assumptions [21, 22]. The multidimensional construct of sensemaking [1, 3, 6] has been demonstrated among HCWs during the COVID-19 pandemic. Hence, HCWs´ individual moral sensemaking in relation to organizational practices was characterized by interaction with others and influenced by external factors and public narratives [23]. Other research on sensemaking in healthcare has focused on various aspects of HCWs´ work during the COVID-19 pandemic. For example, sensemaking has been studied in relation to traumatic stress and resilience when caring for COVID-19 patients [24], job crafting and improvisation [25], resilience and continuity of care among physicians and at-risk patients [26], moral dilemmas with particular attention to collective sensemaking and ethical suffering [27] and redeployment to intensive care units [28, 29]. In short, these studies illuminate how HCWs made sense of the situation by using previous experiences and knowledge to adapt to the present situation [25, 26]. During this time, interaction with others was essential and HCWs made sense of the pandemic together with colleagues through exchange of information and mutual support [24–27, 29]. Furthermore, identification with one´s organisation appeared to play an important role in HCWs´ sensemaking during the pandemic. One example of this was a belief that their organisation was doing everything it could in the context of external pressures [28]. The present study aims to further contribute to the existing literature by explicitly examining sensemaking related to organisational structures, interpersonal interaction and individual experiences within the same empirical study during the early phase of the COVID-19 pandemic. Sensemaking is important in times of crisis because the less adequate the sensemaking, the more likely it is that the crisis will become unmanageable [1]. This study was therefore initiated to gain better understanding of how HCWs understood and managed their work through the perspective of sensemaking during the COVID-19 pandemic.

## Method

### Aim

The aim of the present study is to explore how HCWs managed their daily work during the early weeks of the COVID-19 pandemic through the perspective of sensemaking across organisational, interpersonal and individual levels.

### Study design

A qualitative research approach was chosen to fulfil the aim of the study, which was considered appropriate as interpretation and analysis of non-numeric data enables exploration of deeper meanings of individual experiences and behaviours [30]. Furthermore, individual interviews were used to collect data due to the flexibility they allow in adjusting and refining questions based on participants’ responses, and thereby enhancing the depth and quality of the data [31].

### Setting and participants

The data material used in this study was derived from a larger collection of data including 33 participants in various professions across the Swedish health and social care sector (including physicians, nurses, assistant nurses and administrative staff). These participants were recruited via social media and Swedish healthcare journals. To be included, the participants needed to have had experience of working during the COVID-19 pandemic, and this study focuses on nurses’ and physicians´ experiences. In total 18 participants were included for analysis in this study (12 nurses and 6 physicians), 10 of whom worked in hospital-based care (e.g., emergency care and inpatient wards), and 8 of whom worked in outpatient care (e.g., primary care units and residential care homes). Participants from both urban and rural areas in Sweden were included. Personal questions such as gender and age were not asked during the interviews. However, based on the conversations both men and women participated, and most participants were experienced as female and middle-aged.

### Data collection

Interviews were conducted between 16th March and 5th May 2020 and conducted via video conference (Microsoft Teams), without video camera, or by phone by the last author. At the beginning of each interview, the interviewer explicitly asked the participant whether they were in a setting where the conversation could take place without being overheard or interrupted. Participants confirmed that this was the case; in one instance, the participant moved to a separate room and closed the door to ensure privacy. The participants agreed to their conversation being audio recorded and the interviews lasted between 38 and 94 min with an average of 62 min. The audio-recorded interviews were later transcribed verbatim by a professional transcription company.

The interview guide questions that formed the basis of the analysis included a question whereby participants were asked to describe their most recent full workday, in detail, moment by moment. Other questions used in the analysis captured multiple dimensions of the work context, such as the organisational, informational and relational aspects of work, as well as perceptions of risks. The interview guide (Supplementary File 1) was developed within the broader research project from which the data for the present study were drawn. The interview guide was not designed specifically for this study, as the interview material has also contributed to other publications from the same research project. These publications, written in Swedish, address the significance of infectious disease transmission for work in the health and social care sector during the pandemic [32], informal care and support in the absence of relatives in the patient care [33], and narratives concerning the need for improved working conditions, related to experiences of working conditions before, during, and after the COVID-19 pandemic [34].

### Data analysis

The accounts of the 18 participants were analysed through a reflexive thematic analysis as described by Braun and Clarke [35]. This analysis method was considered appropriate as it enables identification and interpretation of meaning in qualitative data [35], which is in line with the aim of the research. In accordance with reflexive thematic analysis, the analytic process was characterised by the researchers’ subjectivity as an analytic resource, and a reflexive engagement with theory, data and interpretation [36]. The analysis followed these six phases: familiarisation with the data, coding, generating initial themes, developing and reviewing themes, naming themes and writing up [35]. In this study, an existing theory was used to develop the interpretation of the data [35]. More specifically, the perspective of sensemaking across organisational, interpersonal and individual levels was the analytical lens that guided the analysis [4, 11].

First the transcribed interviews were read carefully to gain a comprehensive understanding of the content (phase 1: familiarisation). In the second phase, all transcripts were read once again, identifying segments of data that were relevant to the aim of the research, and the three levels of sensemaking. These segments were assigned coding labels, thus reflecting analytic engagement with the data rather than merely summarising or reducing the content. As a way of structuring the analysis, these codes were sorted into the three analytically informed domains capturing the study´s focus on sensemaking at organisational, interpersonal and individual levels in relation to how the participants managed their daily work during the early weeks of the COVID-19 pandemic. The codes were sorted under the organisational level when statements concerned how the situation was understood through organisational structures. When participants’ accounts included how they made sense of the situation in social interactions, the codes were grouped under the interpersonal sensemaking domain. Finally, the codes were sorted to the domain of individual sensemaking when the participants’ accounts captured their individual thoughts, beliefs and intentions in relation to how they managed their daily work.

The subsequent analysis was developed inductively. For each domain, a shared pattern of meaning was identified whereby clusters of codes that seemed to share a core idea were compiled (phase 3: generating initial themes). Through an active process, initial themes and sub-themes were constructed around the data. In line with Braun and Clarke [35], codes and themes did not ´emerge´ from the data. Rather, the first author played an active role in interpreting codes and themes, and whether they contributed in a relevant way to achieving the aim of the research. In phase 4, these themes and sub-themes were developed and reviewed which included checking that they made sense in relation to the coded extracts as well as the full data set. An important aspect of this phase was ensuring that each theme and sub-theme highlighted the most important patterns related to the aim of the research. The analysis was reviewed through discussions between all authors and fine-tuned by refining, defining and naming the themes and sub-themes (phase 5). Writing was an integral part of the analytic process and started early on, but in the sixth and final step the analytic narratives were weaved together into a coherent story.

The NVivo qualitative analysis package (version 15) was used for conducting the analysis.

### Ethics

The participants received information about the study and data collection via a recruitment page on a Swedish university’s website. This website included research participant information that was designed in line with national and international standards, covering details on information provision, informed consent, confidentiality, and use of research data [37]. In the written information (that was later repeated verbally) participants were informed that study participation was voluntary and they were assured that they could withdraw from the study whenever they wanted, without further explanation. They were assured of confidentiality and that the data would be handled and stored in accordance with GDPR [38] and the Swedish Act on Ethical Review of Research Involving Humans (SFS2003:460) [39]. During the interviews, the interviewer remained attentive to verbal and auditory indications of discomfort, such as changes in tone of voice, prolonged pauses, hesitation, or participants explicitly expressing discomfort. Had any such indications occurred, the interviewer would have paused or discontinued the interview as appropriate. No such indications were noted during the interviews. No participant withdrew their participation during or after the interviews.

To support trustworthiness, the study has been reported according to the COREQ 32-item checklist for qualitative studies [40].

## Findings

The overarching theme, themes and sub-themes developed through the analysis are illustrated in Table 1. The overarching theme **´Sensemaking as a way of holding clinical practice together throughout uncertainty´**, is present throughout the themes and sub-themes, and each one of them reflects a distinct aspect of how clinical practice were held together. Sensemaking across organisational, interpersonal and individual levels include how organisational structures to some extent contributed to stability in the organisation of work, how the situation was understood and handled together and in relation to others, and the capability and commitment of the HCWs to continue to provide care.

**Table 1:** Overview of the overarching theme, themes and sub-themes

Level of sensemaking	OVERARCHING THEME: Sensemaking as a way of holding clinical practice together throughout uncertainty
*Organisational level*	**THEME**: **Organisational directives as boundaries for clinical practice**
*Interpersonal level*	**THEME**: **Relational positioning to manage uncertainty**
	**Sub-theme**: Dealing with uncertainty together
	**Sub-them**e: Compassion towards others
*Individual level*	**THEME: Sustaining care in the face of uncertainty**
	**Sub-theme**: Interpret and understand the situation through past experiences
	**Sub-theme**: Commitment to continuing to provide care despite uncertainty
	**Sub-theme**: Balancing authoritative directives and practical reality

### Theme: Organisational directives as boundaries for clinical practice

At an organisational level of sensemaking, this theme captures how organisational directives created a possibility for stability at work during the early weeks of the COVID-19 pandemic. The analysis indicates that guidelines and recommendations from authorities, above all the Public Health Agency of Sweden and healthcare management, were perceived as creating stabilising boundaries for medical practice. Boundaries from these authoritative sources included classifying patients into risk groups, as they were more likely to be severely affected if exposed to infection in healthcare settings. One HCW illustrated this by saying: *We have primarily used the Public Health Agency’s definition of who belongs to risk groups. Older people with underlying health conditions. That is… well*,* if you have a compromised immune system (…). So we’ve used that as a basis* (IP 4, nurse).

Such guidance also included which patients should be prioritised for care, banning hospital visitors (e.g., relatives), which patients should be tested for COVID-19, and guidelines regarding hygiene routines and personal protective equipment. Thus, the analysis indicates that adherence to the directives and guidelines of the authorities constituted a form of legitimation for their decisions and actions in practice. One HCW described this form of legitimation by exemplifying a conversation with a patient who wanted to be tested for COVID-19: *Then you explain that*,* well*,* this is how it is. We are following all the guidelines from the Public Health Agency*,* and at the moment only such and such people are being tested* (IP 10, nurse).

However, guidelines and directives issued by authorities were provisional and subject to frequent revision, sometimes changing from one day to the next. One HCW illustrated how directives on the use of personal protective equipment requirements changed:

But it’s a bit so-so… they are constantly being updated, and there’s a lot of back-and-forth about what you should and shouldn’t wear, how much protective equipment you need, and how much you should be wearing. At the start, it was just long-sleeved aprons and gloves – maybe even double gloves – and always a face shield. And now it’s more like, ‘Well, no, I think ordinary aprons will do, and yes, maybe without gloves as well’ (IP 7, nurse)

This indicates that the uncertainty of the situation was mirrored in the authorities´ immediate yet continuously evolving decisions. Hence, while organisational directives to some degree stabilised understandings of clinical practice, they were continuously reshaped as decisions evolved.

### Theme: Relational positioning to manage uncertainty

At an interpersonal level of sensemaking, this theme captures how management of the uncertain circumstances was grounded in a collective understanding whereby HCWs in their daily practice reflected together and in relation to others.

#### Sub-theme: Dealing with uncertainty together

It was evident from the HCWs’ accounts that the rapidly changing conditions brought about by the pandemic prompted various thoughts and concerns, and it became clear that the situation could not be understood alone. Instead, through shared engagement on how they should handle the pandemic in practice, discussions with colleagues became an integral part of their work. In these discussions, understanding, attitudes, and choices of action were synchronized. As one HCW noted:

We talked through how to do things, and some people have been a bit more willing to allow the patient to come into the clinic, whilst others have been more stubborn – myself included (…). We simply agreed that we need to do things in pretty much the same way (IP 6, physician)

Thus, connecting with each other in collegial discussions established a shared basis for action whereby the HCWs discussed, for instance, how to adapt patient care according to the perceived risk of infection, and how directives from authorities should be interpreted into clinical practice and the organisation of work in general. One HCW illustrated this as follows:

There’s a sort of stability within the group that makes me feel secure. And they sort out the tasks amongst themselves and sort of take on different roles, which means that we as a group can get our bearings very quickly (IP 2, physician)

This collegial approach did not only arise through verbal discussions, it was also of a more practical nature, where helping each other in work was a necessity. One HCW described how colleagues assisted each other between units: *At the same time as all this*,* we try to support and help one another on the other side… There was a lot going on over there too* (IP 18, nurse). As a whole, this collaboration provided a sense of stability and contributed to HCWs´ ability to orient themselves through the uncertainty.

#### Sub-theme: Compassion towards others

HCWs´ accounts suggested that they interpreted the situation by reflecting on how others were more severely affected by the pandemic. These reflections fostered feelings of compassion for their fellow humans. Although some expressed concerns about their own health in relation to the pandemic, the accounts predominantly show that their concerns were more related to others. More specifically, they appeared more worried about the potential consequences of infection for others (e.g., patients, colleagues, relatives in risk groups, or society at large) than for themselves. This way of caring for others was illustrated by a HCW who noted:

And I was supposed to be working alongside an assistant nurse, but he… it was his first day back after… he’d just had his second child. So we rearranged things so that he could be on the… well, the clean side… so that he wouldn’t have to… We didn’t want him to be among patients with COVID at the time (IP 18, nurse)

Regarding their work situation generally, some of them accepted and made sense of their own situation by positioning themselves in relation to the fact that other parts of the healthcare system were under greater strain due to the pandemic. This approach was reflected in that the distribution of personal protective equipment was perceived to be reasonably prioritised in relation to the needs of their fellow humans.

HCWs´ accounts also included a wish that more severely affected units received sufficient resources to manage the situation. One HCW said:

And then I feel, I’m not really that worried about us. I’m more worried about my colleagues at the hospital, A&E, the ICU, the wards. But mainly A&E, the ICU and… well, the infection ward, which is… well, which is having a tough time. I can sense that a bit. Even though I know they’ll manage it, goodness me, but you do feel a bit concerned for your colleagues, that they’ll get… We’re not exposed to any infection, but they are (IP 10, nurse)

The analysis thus indicates that HCWs viewed, their fellow humans compassionately in the difficulties that working during the pandemic entailed. As such, their understanding of the situation was shaped by how others were affected.

Expressions of concern for others were also reflected in the opposite direction, as some HCWs discussed how they received greater appreciation from the public. One HCW reflected on this by saying: *Because we’re getting a really positive response from the community at the moment. And obviously*,* that’s motivating to a certain extent*,* and it makes you happy* (IP 12, physician). This quote thus reflects how their work situation and efforts were recognised and gave them a sense of meaning.

### Theme: Sustaining care in the face of uncertainty

At an individual level of sensemaking this theme captures the HCWs´ capability and commitment to continuing to provide care, despite the uncertain circumstances.

#### Sub-theme: Interpret and understand the situation through past experiences

The analysis shows that the uncertain circumstances prompted attempts to construct plausible interpretations based on previous experiences. The situation was highlighted by some HCWs as to some extent resembling what they had previously experienced, although the pandemic as a phenomenon was unknown. One HCW expressed this as follows: *We are used to being aware of*,* or rather*,* taking control of threats and risks* (IP 5, nurse).

Another HCW similarly noted:

We do, after all, have patients with highly contagious and dangerous bacteria in other cases as well. We have patients whose lungs are full of resistant MRSA and … well, and so on. So yes, I suppose we’re quite used to this (IP 9, nurse)

The analysis thus suggests that relating to previous experiences may have contributed to a sense of confidence in the present and was shown in certain aspects of their work. For example, due to previous experiences of working in high-pressure environments, such as emergency departments, HCWs described that they were used to navigating rapidly changing situations. One HCW said: *I’m fairly used to making changes*,* having worked in A&E. I’m probably not really the type to stick to a strict routine* (IP 14, nurse).

Previous experiences of providing palliative care, or other critical care, were also mentioned as supporting a professional approach and confidence in the care of severely ill COVID-19 patients.

#### Sub-theme: Commitment to continuing to provide care despite uncertainty

This sub-theme captures a commitment to maintaining care characterised by the recognition that healthcare could not be put on pause. One HCW expressed this by saying:

But just because a new virus has entered the community that we need to deal with urgently, that doesn’t mean that all the other problems the healthcare system is constantly struggling and grappling with every day … well, they don’t just disappear (IP 12, physician)

The analysis reflects that uncertainty was not perceived as a paralysing factor and was instead managed through present-focused actions. The HCWs described how they were able to quickly reorganise the provision of care through adaptive strategies, while simultaneously minimising the risk of spreading infection. Consequently, HCWs managed the uncertain circumstances partly through a short-term focus, to take things day by day, sometimes hour by hour. One HCW said:

Then I get out my to-do list and that’s when… The thing about the current situation with corona is that, first of all, you focus on the next few hours. What needs to be done to make sure that this day runs smoothly? (IP 4, nurse)

Depending on where the HCWs worked, their experiences ranged from it being a rather acceptable and undramatic situation to one characterised by chaos. Although they were all concerned to various degrees, gradual acceptance emerged from their accounts, including a commitment to continuing their work. Two HCWs illustrated different nuances on this stance as follows:

I’ve found it really fascinating to be part of this critical phase, you know. And trying to figure out what we need to do within our department to make sure things turn out well here (IP 15, nurse)

It’s actually… I mean, it’s a scary virus, but you can’t just go around being afraid. That’s how I see it. We just have to do what we’re supposed to do. (…). There’s no reason why we shouldn’t work with a suspected case of COVID, but… and that’s when I think to myself that we simply have to protect ourselves and do our best (IP 13, nurse)

Furthermore, in striving to maintain care, they also oriented themselves towards the future. This orientation included anticipating possible scenarios and preparing for a different and potentially worsening future. One HCW reflected on this by saying: I think it’s going to be really tough. But somehow I feel like I’ve prepared myself for the fact that it’s going to be really hard (IP 11, physician).

This forward-looking orientation was apparent in their descriptions of how they, for instance, secured access to personal protective equipment, as well as in concerns about future high levels of staff sick leave, and that postponed aspects of care would need to be addressed in the future. As a whole, the analysis indicates that maintaining care through continuous orientation in the present, but mindful of the future was essential for the HCWs. In this regard, HCWs constructed meaning through an ongoing effort to provide care, despite circumstances of uncertainty.

#### Sub-theme: Balancing authoritative directives and practical reality

While directives at the organisational level of sensemaking functioned as stabilising boundaries for clinical practice (the first theme), this sub-theme captures how HCWs interpreted directives and guidelines from authorities in order to navigate clinical work during the pandemic. The analysis shows that following these directives and remaining updated could give somewhat of a sense of safety in professional practice. Some examples included the use of personal protective equipment and following established routines to prevent the spread of infection. Yet adherence to these directives was sometimes perceived as complicating daily clinical practice by creating general strain and frustration, and sometimes also made it difficult to fully prevent the spread of infection in practice. Hence, tension sometimes existed between authorities and how things worked in practical reality. One HCW exemplified this by saying:

Well, those at the top are unlikely to know what it’s really like on the ground. So that’s where you’d hope they’d go out to the various departments, do an inspection, have a look around, ask questions, and show a bit of curiosity. How do you feel about this? Show us who you are *(*IP 13, nurse)

Accordingly, the analysis indicates that HCWs positioned their own professional values in practice as a counterbalance to established directives. This point was captured in the following account: *I’m in the thick of it*,* in the midst of a disaster (…)*,* I have to look after myself; that’s what being professional is all about; I’m not going to put up with just anything* (IP 16, nurse). This reflects an awareness of the HCWs´ right to safety in their work, which contradicts the perception that there was largely a shortage of personal protective equipment across different sectors of the healthcare system. Furthermore, the analysis suggests that directives from authorities were sometimes inconsistent with HCWs´ professional values regarding appropriate care provision. These professional values also served as a moral compass. This was partly evident in the fact that some HCWs defied directives prohibiting relatives from visiting patients, as these directives conflicted with their understanding of appropriate professional conduct and patient care. One HCW exemplified this by saying:

One night I actually let a relative come in who was perfectly healthy, because the patient was in such a bad way and so worried and… So we bend the rules a bit when we see that, well, no, we hardly had any patients at the time, and we said, “No, for the sake of that patient, relatives are allowed to come in.” But otherwise, it’s us who say, “Sorry, we’re not allowed to. Sorry.” And it’s really hard having to say that (IP 13, nurse)

In a similar fashion, professional and moral values sometimes guided actions away from strict interpretation of directives when staff performed patient consultations, for example if people were in risk groups due to age, but were otherwise healthy, or when the correct personal protective equipment was not used in life-threatening situations. One HCW illustrated this point by saying:

And also if it’s a child who isn’t breathing and I’ve only got an apron and gloves, well, then we just have to get on with it. I mean, it’s not as if I’m going to stand there and get dressed first and waste precious seconds (IP 15, nurse)

Overall, HCWs considered both directives from authorities and their professional and moral values, when making sense of the uncertain situation, and how they should act in practice. The accounts point to a tension between what worked in theory in terms of directives from authorities, and how this was implemented in practical reality.

## Discussion

This study explored how HCWs managed their daily work during the early weeks of the COVID-19 pandemic through the perspective of sensemaking across organisational, interpersonal and individual levels. This study reveals that clear organisational directives, relational positioning and individual capability and commitment are key factors of sensemaking that create stability and sustainability of work in times of crisis.

At an organisational level, this study shows that directives from authorities legitimised the decisions that HCWs made and actions they took in practice. This reflects how the organisation provided a framework that guided them on how to interpret and act upon the given situation [11]. Thus, organisational structures can contribute to sensemaking during a workplace crisis, creating a shared set of expectations about how the situation should be understood and what to be done [3]. Hence, our findings suggest that adherence to COVID-19-related directives from authorities to some degree helped HCWs to orient themselves in the new situation and hold clinical practice together. However, given the continuously evolving directives on how to handle the pandemic, as well as changing working conditions in general, a continuous reinterpretation of the situation was needed which reflects how sensemaking is dynamic [4, 8] and develops in response to prevailing conditions. Accordingly, in order to hold clinical practice together, the situation was managed in interactions with colleagues, which for instance included how these authoritative directives were interpreted together in relation to practical reality. In line with previous research on sensemaking by HCWs during the COVID-19 pandemic [24–27, 29], HCWs´ accounts clearly highlight how they interacted with colleagues to orient themselves through the uncertainty. This can be understood in relation to the literature highlighting that sensemaking is a social construction [4] whereby communication and collegial discussions are essential for orientation in new situations [6, 8, 9]. Our findings indicate that the interaction between colleagues, constructed a sense of stability under the uncertain circumstances that prevailed. Consequently, these intersubjective meanings created a more orderly environment by fostering a shared understanding that guided coordinated actions [6].

Another notable finding regarding interpersonal sensemaking was that HCWs interpreted the situation by reflecting on how others may be more severely affected by the pandemic. This can be understood through how people construct meaning by interpreting the experiences of others [9]. These findings suggest that HCWs´ compassion for the situations of others, such as HCWs working in other units, helped them to understand and accept their own.

Nonetheless, HCWs did not face the pandemic completely unequipped, and consistent with previous research [25, 26] they made sense of what was going on based on their past individual experiences. This finding reflects that sensemaking is about plausibility rather than accuracy, meaning that individuals make interpretations that are sufficiently coherent rather than objectively correct [4]. The analysis indicates that by using previous individual experiences, HCWs tried to make a plausible interpretation of how to handle the uncertain circumstances that prevailed.

The desire to hold clinical practice together was also reflected in the HCWs´ capability and commitment to continuing to provide care day by day, as well as their thinking about how care should be provided in the unknown future. This highlights how sensemaking is about linking the past, present and future, and thereby providing structure in uncertain situations [8]. In this regard, the findings suggest that HCWs had an action-oriented approach to the uncertain circumstances that prevailed. This is consistent with previous research showing that care still needed to be provided during the pandemic, although often through improvisation [25, 26]. Related to the perspective of sensemaking, this action-oriented approach can be understood through enactment whereby people continuously structured and shaped the environment in which they operated [1, 4]. These findings imply that HCWs shaped a reality in which their efforts to hold clinical practice together became a part of their sensemaking. However, in their commitment to providing care, HCWs also considered whether authoritative directives were reasonable in relation to clinical work, and sometimes they defied them through their actions. As such, a tension between authoritative directives and clinical work existed, and through development of plausible meanings some cues from the environment were selected, while others were neglected [8]. The findings thus reflect that certain cues such as the patient´s immediate needs were sometimes prioritised, while more general directives were de-prioritised. Such experiences may be understood in the light of moral decision making in which individuals tend to choose the option that produces the most favourable consequences rather than opting for strict adherence to specific rules [41]. Accordingly, related to our findings, it is suggested that HCWs demonstrated interpersonal understanding and empathy grounded in moral considerations toward their patients, which at times outweighed adherence to formal directives. However, it is worth noting that addressing patient’s needs during the COVID-19 pandemic also may cause moral distress and the compromise of HCWs´ moral integrity [42, 43]. Consistent with our findings, previous research has reported tensions between pandemic-related policies and patients’ needs, such as visitor restrictions for palliative care patients [43], which highlights the importance of integrating infection prevention and control logic with relational ethics [43].

Our findings of sensemaking, contributing to the maintenance of holding clinical practice together during a highly disruptive period, can be reflected upon in relation to resilience. The concept of resilience captures how an actor (e.g., an individual or organisation) develops and mobilizes capabilities when adapting to environmental demands and sustaining effective functioning before, during and after adversity [44]. It has been shown that resilience is an important attribute in healthcare organisations and can be manifested among professionals in disaster medicine management systems. For example, among these professionals resilience can be identified in their ability to make quick adaptions to current plans, the use of previous strategies that have been successful, and recognizing employees´ competencies as a valuable resource in crisis management [45]. These strategies can be reflected in relation to our findings where HCWs strived to sustain care, for instance through adaptive strategies and use of previous knowledge and experiences. Sensemaking has been recognized as a factor influencing resilience [46]. Although resilience was not explored in this study, it is possible that HCWs through their sensemaking displayed characteristics associated with resilience.

### Strengths and limitations

One strength of this study is the timing, i.e., that the interviews took place right at the beginning of the COVID-19 crisis thereby capturing accounts produced in the immediate aftermath of participants’ experiences, and not through retrospective constructions. This may have otherwise represented a challenge in data collection, as participants´ responses are dependent on their ability to recall past events [47].

Another strength is the open structure of the interview guide, which enabled participants to tell their own stories and facilitated rich and in-depth accounts [31]. Likewise, the study highlighted shared interpretations of working in healthcare teams during a crisis rather than exploring possible differences between different groups of HCWs or types of workplace settings. Accordingly, the inclusion of both nurses and physicians working in different sectors strengthened the study. A heterogenous sample enabled a wide range of perspectives and experiences, which is preferable in qualitative research [48].

To enhance trustworthiness of the study, the research process was characterized by a reflective and thoughtful engagement with the data and the analytic process [49]. In more concrete terms this process captured a striving for conceptual coherence where the different elements of the research design, such as theoretical assumptions, research aim, analysis and findings were in conceptual alignment [35]. Through continuous discussions between the authors, it was ensured that the analysis was related to the research aim and that the development of themes was grounded in the empirical data, as well as within the theoretical perspective of sensemaking.

The authors behind the study have various backgrounds in physiotherapy, public health science, occupational health science, nursing, medical biology and sociology. Authors have extensive experience of research within the healthcare sector and nursing sciences, as well as clinical experience of working in healthcare. These preconceptions in terms of previous experiences and interest in what is to be studied [50] were present throughout the analysis and interpretation of the data. Since the pandemic ended several years ago, it is important to consider potential influence of retrospective interpretation and authors’ own experiences of the pandemic throughout the analysis process. First of all, although authors have clinical experiences of working within different healthcare organizations, none of them worked in a clinical setting during the COVID-19 pandemic. This may have resulted in none to minimal influence of author’s own experiences on data interpretation. Secondly, the potential influence of retrospective interpretation was addressed by repeatedly returning to the original interview transcripts and interpreting participants’ accounts in relation to the specific context in which they were generated, i.e., during early weeks of the COVID-19 pandemic. The interviews and transcripts were also reviewed by the last author during and relatively soon after the data collection period, which supported familiarity with the original context of the participants’ accounts. Nevertheless, the authors acknowledge that subsequent knowledge and experiences of the pandemic may have influenced the later interpretation of the data, and this possibility was considered reflexively throughout the analytical process.

This study also has its limitations, in that the findings are not transferable to the whole healthcare setting in Sweden. Another limitation is that participants were recruited through social media. Recruitment through social media might have a negative impact on the representativeness of the sample as certain groups are more likely to use these platforms [51]. Furthermore, we analyzed HCWs´ accounts through three levels of sensemaking, but not sensemaking as a process and how the different levels of sensemaking interacted with one another. Consequently, the present study does not allow for conclusions to be drawn regarding the nature of the interaction between levels of sensemaking.

In this study the criteria of information power were used to guide sample size [52], i.e., the more information of relevance for the study, the fewer interviews needed. The size of a sample with sufficient information power is dependent on the aim, sample specificity, use of established theory, quality of the dialogue of the interviews and use of analysis strategy [52]. The aim of the study was relatively narrow, focusing on sensemaking during the pandemic, and not covering all work aspects of HCWs. A broader study aim would have required a larger sample size [52]. The sample was heterogenous and can be considered to have a relatively sparse sample specificity which implies that more interviews might be needed [52]. However, the dialogue with HCWs was strong and provided rich accounts with respect to the aim of the study. This means that fewer interviews are required to provide sufficient information power when the communication between the researcher and participant is strong and clear [52]. One final point to consider is that the study was supported by established theory guiding the data analysis, which indicates that fewer interviews are required [52]. As a whole, these factors contributed to adequate information power, indicating that 18 interviews were reasonable.

## Implications for clinical practice

The findings of this study have implications for clinical practice, particularly regarding preparedness for future healthcare crisis and other forms of uncertainty. Findings imply that sensemaking at the organisational, interpersonal, and individual level are an important consideration for healthcare organisations when facing future crisis or other uncertain events. Regarding sensemaking at the organisational level, clear organisational directives are important for fostering a shared understanding of how uncertain situations should be interpreted and managed in practice. Furthermore, HCWs need opportunities and time for interaction with colleagues, enabling them to collectively construct meaning and maintain a sense of stability when managing uncertainty (interpersonal sensemaking). It is also important to acknowledge the individual HCWs´ sensemaking, which are shaped by their individual knowledge, experiences and capability to sustain clinical practice despite conditions of uncertainty.

## Conclusions

This study provides insight into how HCWs engaged in sensemaking across organisational, interpersonal and individual levels during the uncertain conditions that prevailed during the early weeks of the COVID-19 pandemic. Sensemaking across the levels was characterised by a pattern of holding clinical practice together. This was apparent in how the situation was understood through stabilising organisational structures that legitimised actions and decisions, while relational positioning constituted a sense of stability and acceptance of their own situation. Furthermore, HCWs´ sensemaking was highly characterised by an individual capability and commitment to continue providing care despite the uncertainty posed by the pandemic. Even though the COVID-19 pandemic is now more or less a closed chapter from a risk perspective, future crises are likely to occur. By focusing on crisis as a phenomenon this may provide a holistic understanding of sensemaking by healthcare professionals in such events. However, this area needs to be studied further, and we suggest that future research explores how sensemaking can be understood at organisational, interpersonal and individual levels in crises or other acute events that affect healthcare workplaces and other contexts.

## Supplementary Information

Below is the link to the electronic supplementary material.

Supplementary Material 1

## Data Availability

The data material analysed in this study is not publicly available. Participants have consented to the publication of aggregated data, but not to open publication of data for individuals. Data is available from the corresponding author upon reasonable request.
