# Supplementary material for: How Swedish healthcare workers managed the early weeks of the COVID-19 pandemic: sensemaking across organisational, interpersonal and individual levels
Source: BMC Health Serv Res. 2026 Oct 10;26:1395. doi: 10.1186/s12913-026-15826-y (PMC13660175; doi:10.1186/s12913-026-15826-y)
Supplement: Supplementary file 1 [file 12913_2026_15826_MOESM1_ESM.docx]

# Interview guide

**Information**

The participants are provided with information about the study. The participants are being asked whether they have read the written information about the study, and whether they give their informed consent to participate. They are informed that participation is voluntary and their right to withdraw from the study at any time without giving a reason. Participants are asked if it is okay to audio record the interview.

**Introductory questions**

- Can you describe your work and your workplace?
- Can you describe your role and position?
- What type of position do you hold?
- What are your main job duties and responsibilities?
- What are you currently working on?
- Approximately how many colleagues do you have?
- Which professional groups are represented in your workplace?
- What is your educational and professional background, including your education, work experience and experience of working in health care?
- Is there anything else I should know about your specific work situation to better understand the context in which you work?

**Narrative question**

- Please tell me about your most recent working day, from the moment you left home and went to work (if you were at the workplace), through the whole working day, moment by moment, as descriptive and in as much detail as you can, until you closed the door behind you and went home again.

**In-depth questions about the work situation during the initial stages of the pandemic**

- In which aspects of your work is the spread of the Coronavirus particularly important?
- Are there aspects of your work where the spread of the coronavirus is not important, everything is business as usual?
- How do you view the relationship between patients/residents and relatives? The role of relatives?
- Palliative care?
- Are there any specific situations at work that are perceived as particularly risky right now? What do you think of in terms of “risk” during this initial period of the spread of the Coronavirus? For whom is it a risk? Who is involved when uncertainties or risks arise right now? Is there anything that can be done to reduce these uncertainties and/or risks? If so, who can contribute?
- How is information managed during this period? What information do you receive, from whom, and through which channels? What are the conditions for receiving and handling information?
- Are there people/functions in your work that can help you navigate your work during this new situation with the spread of the Coronavirus? If so, who?
- Are there people, organizations or authorities that you trust more or less regarding this situation?
- What does the employer need to know about your work situation in order to better provide support during this situation?
- What does the authorities need to know about your work situation in order to better provide support during this situation?

**Closing question**

- Any other thoughts about your work situation during this initial period of the spread of the coronavirus that have come up? Anything I missed to ask about?
- How did you feel about doing this interview?
